# Assessment of Human Epididymis Protein 4 Expression in Breast Ductal Carcinoma In Situ †

**DOI:** 10.3390/diagnostics15091058

**Published:** 2025-04-22

**Authors:** Nah Ihm Kim, Min Ho Park, Ji Shin Lee

**Affiliations:** 1 Department of Pathology, Chonnam National University Medical School, Gwangju 61469, Republic of Korea; mince1234@naver.com; 2 Department of Surgery, Chonnam National University Medical School, Gwangju 61469, Republic of Korea; mhpark@chonnam.ac.kr

**Keywords:** ductal carcinoma in situ, breast, HE4, serum, mRNA, protein, biomarker

## Abstract

**Background/Objectives**: Elevated expression of human epididymis protein 4 (HE4) has been observed in breast cancer and is associated with cancer progression; however, its role in ductal carcinoma in situ (DCIS) remains unclear. This study aimed to evaluate HE4 levels in serum and tissue from patients with DCIS and their correlation with clinicopathological features. **Methods**: Preoperative serum HE4 levels were measured in 59 DCIS patients. HE4 mRNA and protein expression in DCIS and adjacent normal tissues were assessed using RNAscope in situ hybridization and immunohistochemistry, respectively. An additional independent tissue microarray of 41 DCIS cases was also analyzed for HE4 expression in tumor tissue only. Furthermore, the BreastMark database was applied to assess the prognostic significance of HE4 expression in a larger cohort of breast cancer. **Results**: Serum HE4 levels (mean ± SD: 39.4 ± 11.9 pmol/L) were within the normal range and showed no significant correlation with clinicopathological parameters except menopausal status. HE4 expression was significantly higher in DCIS tissues compared to adjacent normal tissues, with a positive correlation between mRNA and protein levels (*r* = 0.771, *p* < 0.001). High HE4 mRNA and protein expression was associated with ER positivity, HER2 negativity, low stromal tumor-infiltrating lymphocyte density, and HR+/HER2− subtypes, but was not predictive of DCIS recurrence. In breast cancer patients, high HE4 expression was significantly correlated with improved survival outcomes. **Conclusions**: Although serum HE4 is not elevated in DCIS, high HE4 expression in tissue is associated with favorable clinicopathological features. These findings highlight the need for further investigation into the potential prognostic role of HE4.

## 1. Introduction

Ductal carcinoma in situ (DCIS) is a non-obligate precursor of breast cancer characterized by neoplastic proliferation of mammary ductal epithelial cells confined to the ductal–lobular system [1,2]. In Korea, ductal carcinoma in situ (DCIS) accounts for approximately 15% to 20% of newly diagnosed breast cancer cases, with its incidence increasing alongside the widespread implementation of mammographic screening programs [3]. However, mammography has limited sensitivity in younger patients, particularly in detecting small lesions such as DCIS, thereby reducing its diagnostic accuracy in this population [4]. DCIS is a biologically heterogeneous disease with diverse clinical outcomes [1,2]. Consequently, there remains a critical need to identify reliable diagnostic and prognostic biomarkers for detecting DCIS and predicting its recurrence following surgical or adjuvant treatment.

Human epididymis protein 4 (HE4), also known as WFDC2, is a secretory protein belonging to the whey acidic protein (WAP) domain family [5]. It exhibits diverse biological functions, including protease inhibition, and plays a role in the innate immune defense across various epithelial tissues [6]. HE4 is expressed in the normal epithelium of the reproductive tract and the proximal airways [5,6]. Notably, elevated HE4 expression has been reported in several malignancies, particularly gynecologic and pulmonary cancers [7,8,9]. HE4 is recognized as a valuable serum biomarker for the diagnosis of epithelial ovarian cancer and can be used to monitor disease progression and recurrence in affected patients [10,11,12].

Recent studies have demonstrated elevated serum HE4 levels in patients with breast cancer, suggesting its potential utility as a diagnostic biomarker for breast carcinoma [13,14,15,16,17,18,19]. HE4 is also expressed in breast cancer tissues [18,20,21,22], and its expression has been proposed as a potential predictor of disease recurrence [18,21]. However, the expression of HE4 in DCIS and its clinical significance have not yet been fully elucidated.

This study aimed to evaluate serum and tissue levels of HE4 in patients with DCIS and their associations with clinicopathological characteristics. Preoperative serum HE4 concentrations were measured in 59 patients with DCIS. Concurrently, HE4 mRNA and protein expression levels were assessed in DCIS tissues and corresponding adjacent normal breast tissues using RNAscope in situ hybridization (ISH) and immunohistochemistry, respectively. An additional independent cohort comprising 41 DCIS tissue samples was also analyzed for HE4 expression. To further investigate the prognostic significance of HE4, its expression was examined in a broader cohort of breast cancer patients using data from the BreastMark database. This article was partly presented at the 2024 European Congress of Pathology [23].

## 2. Materials and Methods

### 2.1. Serum and Tissue Samples

This study included patients with primary DCIS who underwent surgery between 2005 and 2014 and whose samples were stored in the Chonnam National University Hwasun Hospital (CNUHH) Biobank. Serum samples from 59 patients with DCIS, along with corresponding tissue microarrays containing both DCIS lesions and adjacent normal breast tissues, were obtained from the CNUHH Biobank. The serum samples were collected before surgery and stored in a liquid nitrogen freezer. Tissue microarrays were constructed to include both DCIS lesions and matched adjacent normal breast tissues. From each formalin-fixed, paraffin-embedded (FFPE) DCIS specimen, two to three cores were taken from the tumor area and one to two cores from the adjacent normal tissue, each measuring 2.0 mm in diameter, were obtained based on the regions marked by the pathologists on the slide. Additionally, we obtained a separate set of tissue microarrays containing only DCIS tumor cores from 41 patients, with two to three cores included per case, also sourced from the CNUHH Biobank.

### 2.2. Serum Concentrations of HE4

Serum HE4 levels were measured using chemiluminescent microparticle immunoassays on the fully automated ARCHITECT instrument (Abbott Diagnostics Division, Lake Forest, IL, USA) at the Department of Laboratory Medicine at CNUHH. According to the manufacturer’s guidelines, HE4 levels above 70.4 pmol/L in premenopausal women and above 140.0 pmol/L in postmenopausal women were considered abnormal [12].

### 2.3. RNAscope In Situ Hybridization (ISH) Assay

Chromogenic RNAscope ISH for HE4 mRNA transcripts was performed using the RNAscope FFPE Assay kit (Advanced Cell Diagnostics, Hayward, CA, USA), following previously described protocols [24]. Briefly, serial sections of TMA block slides underwent deparaffinization, dehydration, heat treatment, and protease digestion before hybridization with target probes for human Hs-WFDC2 (Cat# 524781, Advanced Cell Diagnostics), peptidylprolyl isomerase B (Hs-PPIB) as a positive control, and bacterial dihydrodipicolinate reductase (DapB) as a negative control. The hybridization signal was detected using the RNAscope 2.0 HD Reagent kit (Brown) (Advanced Cell Diagnostics) with 3,3′-diaminobenzidine solution as the chromogen. Slides were counterstained using Gill’s hematoxylin.

RNAscope amplification was interpreted semi-quantitatively in accordance with the manufacturer’s guidelines: 0: no staining or <1 dot/10 cells at 40×; 1: 1–3 dots/cell at 20–40×; 2: 4–10 dots/cell at 20–40×; 3: >10 dots/cell with <10% of positive cells having dot clusters visible at 20×; 4: >10 dots/cell with >10% of positive cells having dot clusters at 20×. Slides with PPIB scores of ≥2 and DapB background scores of ≤1 were considered suitable to pass the qualification of tissue mRNA. The HE4 mRNA expression level was classified as low (score: 0–2) or high (score: 3–4) [24].

### 2.4. Immunohistochemistry

HE4 immunohistochemical staining was performed using a Bond-max Autostainer (Leica Microsystems, Bannockburn, IL, USA), as previously described, in conjunction with a HE4 rabbit polyclonal antibody (1:800; Atlas Antibodies, Stockholm, Sweden) [25]. HE4 immunoreactivity was semi-quantitatively assessed based on both cytoplasmic staining intensity and the percentage of positively stained cells, following a previously published scoring method [20]. Staining intensity was scored as follows: 0 (no staining), 1 (weak), 2 (moderate), and 3 (strong). The extent of staining was scored according to the proportion of stained tumor cells: 0 (no positive cells), 1 (1–24%), 2 (25–49%), 3 (50–74%), and 4 (75–100%). The final staining score was calculated by multiplying the intensity and extent scores. For statistical analyses, cases with a final score between 0 and 4 were classified as having low HE4 expression, while those with scores ≥4 were considered to have high HE4 expression [20].

### 2.5. Clinicopathological Characteristics of DCIS Patients

Clinicopathological characteristics, including patient age, menopausal status, tumor size, nuclear grade, comedo-type necrosis, and patient outcomes (local recurrence and survival), were retrieved from medical records. Local recurrence was defined as any in situ or invasive carcinoma recurrence in the ipsilateral breast, axilla, or chest wall. Estrogen receptor (ER), Progesterone receptor (PR), and human epidermal growth factor receptor 2 (HER2) expressions were assessed in all samples. ER or PR positivity was defined as nuclear staining in at least 1% of tumor cells [26]. HER2 positivity was defined as a 3+ score on immunohistochemistry, following the guidelines of the American Society of Clinical Oncology (ASCO)/College of American Pathologists (CAP) [27]. Based on hormone receptor (HR; ER or PR) and HER2 expression, DCIS cells were classified into four subtypes: HR+/HER2−, HR+/HER2+, HR−/HER2+, and triple-negative (ER−/PR−/HER2−).

The density of stromal tumor-infiltrating lymphocytes (TILs) was evaluated as the percentage of tumor stroma occupied by lymphocytic infiltrates, excluding those in direct contact with DCIS cells. TILs were categorized into four groups: 0 (no TILs), 1 (<5% of stromal area with TILs), 2 (5–50% of stromal area with TILs), and 3 (>50% of stromal area with TILs) [28].

### 2.6. HE4 as Prognostic Biomarker in Patients with Breast Cancer Using BreastMark

The prognostic significance of HE4 expression in breast cancer was assessed using the BreastMark database as previously described [29]. BreastMark is an online tool for evaluating the prognostic significance of putative genes in breast cancer. It integrates gene expression and survival data from 26 datasets across 12 different microarray platforms, encompassing approximately 17,000 genes in up to 4738 samples. Among these, data for the gene HE4 (WFDC2) were available in 2652 samples, including 744 lymph node–positive, 1183 lymph node–negative, 823 luminal A, 1013 luminal B, 286 HER2-positive, and 424 basal-like tumors. Disease-free survival (DFS) was analyzed based on HE4 expression, which was dichotomized using the median value as the cutoff. DFS was evaluated for the overall breast cancer cohort, as well as within subgroups stratified by lymph node status (node-positive vs. node-negative) and by molecular subtype, according to the PAM50 classification (luminal A, luminal B, HER2-enriched, and basal types). Kaplan–Meier survival curves were generated to visualize differences in DFS, and statistical significance was assessed using the log-rank test. Cox proportional hazards regression analysis was performed to estimate hazard ratios (HRs) along with their corresponding 95% confidence intervals (CIs)

### 2.7. Statistical Analysis

Statistical analyses were performed using SPSS software (Version 13.5, SPSS Inc., Chicago, IL, USA). Serum HE4 levels were analyzed using the independent t-test, one-way analysis of variance (ANOVA), and Kruskal–Wallis tests. Differences in HE4 mRNA and protein expression between DCIS tissues and adjacent normal breast tissues were evaluated using the Mann–Whitney U test. Spearman’s correlation was used to evaluate the association between variables. Categorical nominal variables were analyzed using the chi-square test or Fisher’s exact test, as appropriate. The linear-by-linear association test was applied to evaluate trends across variables. A *p*-value of less than 0.05 was considered statistically significant.

## 3. Results

### 3.1. HE4 Serum Levels in Patients with DCIS and HE4 mRNA and Protein Expression in DCIS Tissues and Adjacent Normal Breast Tissues

Serum HE4 concentrations were successfully analyzed in all 59 patients with DCIS. The measure HE4 levels ranged from 23.5 to 86.3 pmol/L (mean ± SD: 39.4 ± 11.9). None of the patients exceeded the manufacturer’s recommended cutoff values for abnormal HE4 levels (70.4 pmol/L for premenopausal women and 140.0 pmol/L for postmenopausal women). Serum HE4 levels did not show significant associations with clinicopathological parameters, except for menopausal status (Table 1).

**Figure 1:**
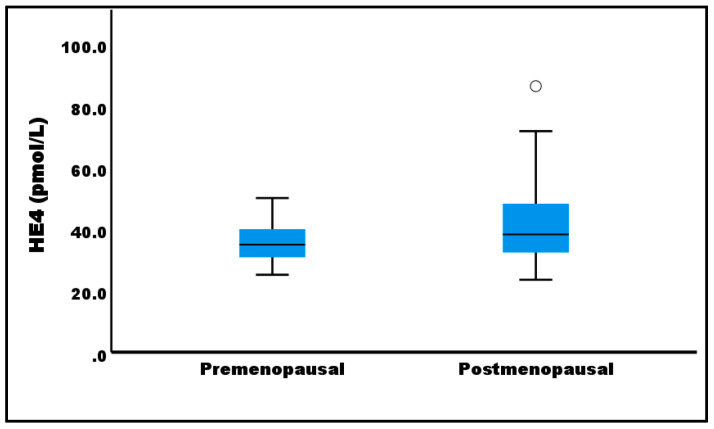
Box plots of serum HE4 levels according to the menopausal status. The whiskers show the maximum and minimum values, with the exception of outliers (circles).

Serum HE4 concentrations were significantly higher in postmenopausal patients with DCIS compared to their premenopausal counterparts (42.9 ± 14.4 pmol/L vs. 35.3 ± 6.4 pmol/L; *p* < 0.05; Figure 1).

**Figure 2:**
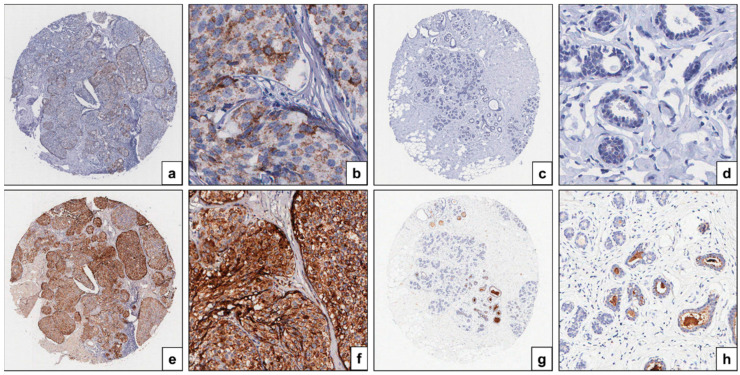
Expression and localization of HE4 mRNA (**a**–**d**) and protein (**e**–**h**) in ductal carcinoma in situ (**a**,**b**,**e**,**f**) and adjacent normal breast tissues (**c**,**d**,**g**,**h**). mRNA and protein expression levels of HE4 are higher in DCIS tissues compared with adjacent normal tissues. (**a**,**c**,**e**,**g**) ×4; (**b**,**d**) ×400; (**f**,**h**) ×200.

**Figure 3:**
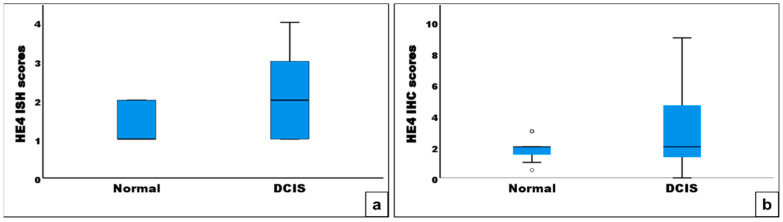
Box plots of HE4 mRNA in situ hybridization (ISH) staining scores (**a**) and immunohistochemical (IHC) staining scores (**b**) in ductal carcinoma in situ (DCIS) and adjacent normal breast tissues. The whiskers show the maximum and minimum values, with the exception of outliers (circles).

ISH and immunohistochemical analyses were performed to examine HE4 mRNA and protein expression in DCIS tissues and adjacent normal breast tissues. Among 59 cases, mRNA ISH and immunohistochemical data were interpretable in 58 cases. HE4 mRNA signals, when present, were primarily detected in the epithelial cells of DCIS lesions (Figure 2a,b), whereas expression was markedly reduced in the epithelial cells of adjacent normal breast tissues (Figure 2c,d). Comparative analysis of HE4 mRNA amplification scores demonstrated significantly higher expression in DCIS tissues than in their matched normal counterparts (1.9 ± 0.9 vs. 1.3 ± 0.5; *p* < 0.001; Figure 3a).

The results of HE4 immunohistochemical staining were consistent with the findings from mRNA ISH analysis. HE4 immunoreactivity was localized to the epithelial compartment in both DCIS and normal breast tissues, with no detectable staining in adjacent stromal cells (Figure 2e–h). In DCIS tissues, HE4 staining was predominantly cytoplasmic (Figure 2e,f). In contrast, the normal breast epithelium exhibited negative to weakly positive staining, while luminal secretions occasionally showed positive immunoreactivity (Figure 2g,h). HE4 immunohistochemical staining scores were significantly higher in DCIS tissues compared to adjacent normal tissues (3.2 ± 2.6 vs. 1.8 ± 0.6; *p* < 0.001; Figure 3b).

Spearman’s correlation analysis revealed no significant association between serum HE4 levels and HE4 expression in DCIS tissues (r = 0.022, *p* = 0.871 for serum vs. mRNA; r = 0.040, *p* = 0.766 for serum vs. protein). In addition, serum HE4 concentrations did not show a significant difference between low and high mRNA/protein expression in DCIS tissues (39.9 ± 12.7 pmol/L vs. 38.9 ± 10.2 pmol/L, *p* = 0.788, for mRNA; 39.8 ± 13.4 pmol/L vs. 39.1 ± 9.1 pmol/L, *p* = 0.838, for protein).

### 3.2. Expression of HE4 mRNA and Protein in DCIS Tissues and Their Correlation with Clinicopathological Features

#### 3.2.1. Clinical Characteristics of the DCIS Patient Cohorts

Among the 100 patients diagnosed with DCIS, 52 were premenopausal and 48 were postmenopausal. Tumor sizes ranged from 0.8 to 10.0 cm in diameter (median: 2.2 cm, mean: 2.8 cm). Most cases were classified as nuclear grade 2 (55 cases, 55.0%) or grade 3 (42 cases, 42.0%). Comedo-type necrosis was identified in 77 cases (77.0%). Stromal TILs were present in all DCIS cases, with 68 cases (68.0%) classified as TIL score 1, 30 cases (30.0%) as score 2, and 2 cases (2.0%) as score 3. Molecular subtyping revealed 48 cases (48.0%) of HR+/HER2−, 17 cases (17.0%) of HR+/HER2+, 29 cases (29.0%) of HR−/HER2+, and 6 cases (6.0%) of the triple-negative subtype. The median follow-up duration was 96 months (range: 7–178 months). During this period, local recurrence occurred in nine patients (9.0%), with no cancer-related deaths reported.

#### 3.2.2. Expression of HE4 mRNA and Protein in DCIS Tissues

Among 100 DCIS tissues analyzed, HE4 mRNA ISH and immunohistochemical staining were interpretable in 99 cases. A significant positive correlation was observed between HE4 mRNA ISH scores and protein immunohistochemistry scores (r = 0.771, *p* < 0.001). Based on predefined cutoff values, high HE4 mRNA expression was identified in 25 cases (25.3%), and high protein expression was detected in 34 cases (34.3%). The concordance between HE4 expression assessed by mRNA ISH and protein immunohistochemistry is summarized in Table 2. An overall concordance rate of 88.9% was observed, indicating a high level of agreement and consistent expression patterns across both methods.

Subsequently, the association between HE4 expression and clinicopathological features in DCIS was examined (Table 3). High HE4 mRNA expression was significantly associated with lower stromal TIL density, ER positivity, HER2 negativity, and the HR+/HER2− subtype. Similarly, high HE4 protein expression correlated with the absence of comedo-type necrosis, lower stromal TIL density, ER positivity, HER2 negativity, and the HR+/HER2− subtype. However, neither HE4 mRNA nor protein expression was significantly associated with DCIS recurrence.

### 3.3. HE4 for Prognostic Biomarker in Patients with Breast Cancer

**Figure 4:**
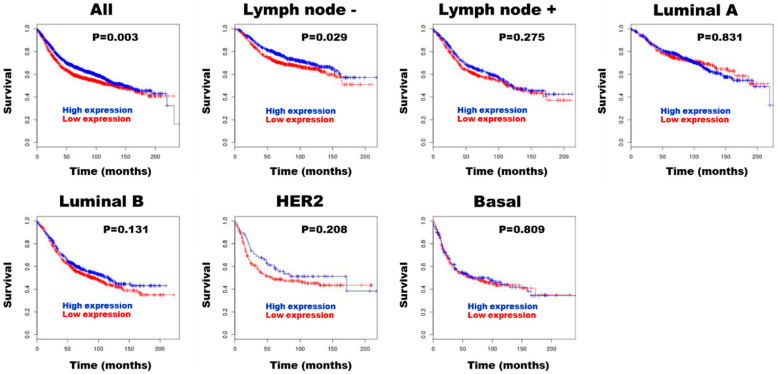
Prognostic role of HE4 expression in breast cancer assessed using BreastMark.

BreastMark analysis revealed that higher HE4 expression was significantly associated with improved survival in both the overall breast cancer cohort (HR = 0.838, 95% CI: 0.75–0.94, *p* = 0.003, *n* = 2652) and the lymph node-negative subgroup (HR = 0.791, 95% CI: 0.64–0.97, *p* = 0.029, *n* = 1183) (Figure 4).

## 4. Discussion

To our knowledge, this is the first study to comprehensively assess both serum and tissue levels of HE4 in patients with DCIS. Although serum HE4 levels remained below the manufacturer-defined cutoff in all cases, high HE4 mRNA and protein expression in DCIS tissues were significantly associated with favorable clinicopathological features.

These findings add a new layer to the existing literature by demonstrating that tissue-level HE4 expression, rather than serum levels, may have clinical relevance in DCIS. This distinction is particularly noteworthy, as prior studies on HE4 in breast cancer have largely focused on invasive carcinomas and relied predominantly on serum-based assays. Our application of both RNAscope in situ hybridization and immunohistochemistry on DCIS samples provides novel insight into the localized expression pattern of HE4 in non-invasive lesions.

DCIS is a non-invasive form of breast cancer, and its incidence has risen considerably with the increased use of mammographic screening. Currently, DCIS accounts for approximately 15–20% of newly diagnosed breast cancer cases in Korea [3]. While mammography remains the standard screening tool, its limitations, particularly in younger women and in detecting small or early-stage lesions, underscore the need for supplementary biomarkers [4]. DCIS is also a heterogeneous disease with varying biological behaviors, ranging from indolent to potentially progressive forms [1,2]. Therefore, identifying reliable diagnostic and prognostic biomarkers for detecting its presence and the risk of recurrence is essential for timely treatment and improved clinical management.

HE4, originally described as an epididymis-specific protein, has gained recognition as a promising biomarker in various malignancies, especially in gynecologic and pulmonary cancers [7,8,9]. Its utility in ovarian cancer is well established. HE4 expression is significantly elevated in epithelial ovarian tumors compared to borderline or benign ovarian lesions, and the protein is actively secreted into circulation [12]. Serum HE4 is now widely used for diagnosis, monitoring of treatment response, and early detection of relapse in epithelial ovarian cancer patients [10,11,12].

In contrast, the clinical utility of HE4 in breast cancer remains less clearly defined. Several studies have examined HE4 expression in both blood and tumor tissues, suggesting its potential as a diagnostic and prognostic biomarker in breast cancer [13,14,15,16,17,18,19,20,21,22]. However, its expression and clinical relevance in DCIS have not been thoroughly characterized.

Most studies have used enzyme-linked immunosorbent assays (ELISA) to measure HE4 levels in the serum or plasma of breast cancer patients [13,14,15,16,17,18,19]. These studies consistently reported significantly higher HE4 levels in patients with breast cancer compared to those with benign breast tumors or healthy controls, supporting its potential role as a diagnostic marker [13,15,16,17,18,19]. However, the lack of standardized cutoff values across studies has limited their comparability and the assessment of diagnostic accuracy. Moreover, few investigations have addressed HE4 expression in non-invasive breast lesions such as DCIS.

In the present study, serum HE4 levels were measured using chemiluminescent microparticle immunoassays on a fully automated ARCHITECT instrument. Among the 59 patients with DCIS, serum HE4 levels ranged from 23.5 to 86.3 pmol/L, with no cases exceeding the manufacturer’s established cutoff thresholds for abnormal levels. These findings suggest that serum HE4 is not elevated in DCIS and, therefore, is unlikely to serve as a useful screening or diagnostic biomarker for this pre-invasive stage of breast disease.

HE4 mRNA and protein expression levels in breast cancer tissues have previously been investigated using real-time PCR and immunohistochemistry [18,20,21,22]. Immunohistochemically, HE4 is generally localized to the cytoplasm of breast cancer cells, and expression has also been reported in normal breast ductal epithelium [20,22]. The reported frequency of HE4 positivity in breast cancer varies widely (9.6–98.8%), depending on the scoring criteria employed [18,20,21,22]. For instance, Mirmohseni Namini et al. observed HE4 positivity in only 3 of 31 (9.6%) breast cancer tissues, with no expression detected in adjacent normal tissues [18]. Several studies have also shown that HE4 mRNA expression is significantly higher in tumor tissues compared to adjacent normal tissues [18,21].

In our study, we evaluated HE4 mRNA and protein expression in DCIS and matched adjacent normal breast tissues using RNAscope in situ hybridization and immunohistochemistry. Both methods demonstrated that HE4 expression was restricted to the epithelial compartment, with no detectable expression in the surrounding stroma. Importantly, HE4 expression levels were significantly higher in DCIS tissues than in adjacent normal epithelium. A strong positive correlation was observed between HE4 mRNA and protein expression levels, suggesting that HE4 upregulation in DCIS may be predominantly regulated at the transcriptional level. These findings support the use of RNAscope ISH and immunohistochemistry as complementary tools for characterizing HE4 expression in tissue-based analyses.

Interestingly, no significant correlation was observed between serum HE4 levels and HE4 expression in DCIS tissues. Serum HE4 concentrations did not significantly differ between cases classified as having low versus high HE4 mRNA or protein expression.

This result is consistent with findings in other malignancies. Previous studies have similarly reported a lack of correlation between serum and tissue HE4 levels in various cancers. For example, no significant association was found between serum HE4 levels and tissue expression detected by immunohistochemistry in endometrial cancer [30,31]. Several factors may account for this discrepancy, including the semi-quantitative nature of tissue expression assessment, which may not capture subtle differences in expression, as well as the inherent heterogeneity of HE4 expression within tumors. It is also possible that the relatively small size and localized nature of DCIS may limit its ability to influence systemic biomarker levels. Although tumor lesions can influence serum biomarker levels, our evaluation of mRNA and protein expression was limited to a small, localized portion of the tumor, representing only a snapshot of overall tumor expression.

The prognostic significance of HE4 in breast cancer remains a subject of ongoing debate. Some studies have reported no correlation between serum HE4 levels and clinicopathological features in breast cancer [14,15]. In contrast, other investigations have identified associations between HE4 overexpression and poor prognostic features, such as higher tumor grade, larger size, advanced stage, and lymph node metastasis [16,18,21,22].

For example, Lu et al. reported significantly higher HE4 levels in stage III disease compared to stage I/II breast cancer [16]. Mirmohseni Namini et al. [18] also found that plasma HE4 levels were significantly associated with tumor grade, stage, and metastatic status.

Moreover, patients with metastatic breast cancer exhibited markedly elevated plasma HE4 levels compared to non-metastatic patients. In the same study, HE4 mRNA expression levels were also significantly correlated with tumor grade in breast cancer patients [18]. In contrast, Kamei et al. evaluated HE4 mRNA expression in breast cancer and found no significant difference in disease-free survival between HE4-positive and HE4-negative groups [21]. They also assessed HE4 expression in breast cancer tissues using immunohistochemistry. No correlation was observed between HE4 expression and clinical parameters, except for lymph node involvement, with HE4 expression being positively associated with nodal metastasis. Although multivariate analysis did not identify HE4 as an independent prognostic factor for disease-free survival, the five-year disease-free survival rate was significantly lower in the HE4-positive group compared to the HE4-negative group. However, median overall survival did not significantly differ based on HE4 staining intensity in breast cancer patients. Taken together, although increased HE4 expression has been associated with adverse clinicopathological features, its relationship to long-term clinical outcomes remains inconclusive.

In DCIS, several clinicopathological features have been associated with a higher risk of local recurrence, including large lesion size, high nuclear grade (grade III), presence of comedonecrosis, positive surgical margins, high stromal TIL density, and HER2-positive or triple-negative molecular subtypes [1,2,32]. In our study, serum HE4 levels in DCIS patients did not differ significantly across clinicopathological variables, except for menopausal status. However, previous studies have reported no association between serum HE4 and menopausal status in breast cancer patients [15,16]. Our findings are consistent with reports in ovarian cancer, where postmenopausal patients exhibited higher HE4 levels than premenopausal patients [12].

High HE4 mRNA and protein expression were identified in 25.3% and 34.3% of DCIS cases, respectively, based on the scoring system proposed by Galgano et al. [20]. Although HE4 expression did not correlate with recurrence in DCIS patients, both mRNA and protein levels were significantly associated with favorable prognostic and predictive features, including absence of comedonecrosis, low stromal TIL density, ER positivity, HER2 negativity, and the HR+/HER2− molecular subtype. These associations suggest that HE4 may serve as a biomarker for biologically indolent forms of DCIS. To further investigate the prognostic potential of HE4 in breast cancer, we conducted a survival analysis using the publicly available BreastMark database. High HE4 mRNA expression was significantly associated with improved survival in both the overall breast cancer cohort and the lymph node-negative subgroup. These findings suggest that HE4 may have a context-dependent role in breast tumor biology, potentially reflecting different functions in non-invasive versus invasive stages. In early-stage disease, such as DCIS, HE4 may be involved in local regulatory or differentiation pathways associated with favorable tumor behavior, whereas in more advanced cancers, its overexpression may reflect or contribute to tumor aggressiveness. Further studies are needed to elucidate the mechanistic role of HE4 in breast cancer progression and to determine whether its prognostic implications differ by disease stage. These results support the hypothesis that high HE4 mRNA and protein expression may serve as a marker of favorable prognosis, not only in invasive breast cancer but also in pre-invasive lesions such as DCIS. Given these findings, we propose that HE4 mRNA and protein expression may be useful as prognostic biomarkers in breast cancer, including DCIS.

However, this study has several limitations that should be acknowledged. First, the sample size of 100 DCIS cases, particularly the relatively small number of recurrence events, may limit the statistical power and generalizability of the findings. Second, although RNAscope in situ hybridization and immunohistochemistry provided complementary insights into HE4 expression, both are semi-quantitative methods and may not fully capture subtle variations or intratumoral heterogeneity. Third, serum HE4 was assessed at a single preoperative time point, limiting our ability to evaluate longitudinal changes or responses to disease progression. Lastly, while our findings suggest biological and clinical relevance for HE4 in DCIS, the study did not include mechanistic or functional assays to directly explore HE4’s role in tumor development or progression. Further studies involving larger, prospectively followed cohorts and incorporating functional investigations are warranted to validate and expand upon these findings.

## 5. Conclusions

This study characterizes HE4 expression in both serum and tissue from patients with DCIS. Serum HE4 levels remained within the normal range and were not elevated above diagnostic thresholds, indicating that serum HE4 is not suitable as a screening or diagnostic biomarker for DCIS. In contrast, high HE4 mRNA and protein expression in DCIS tissues was significantly associated with favorable clinicopathological characteristics. These findings highlight the potential utility of tissue HE4 expression as a prognostic indicator in DCIS, with potential clinical application in risk stratification and treatment planning. Future research should aim to validate these observations in larger cohorts with long-term clinical follow-up and to further explore the biological role of HE4 in early breast carcinogenesis.

## Figures and Tables

**Table 1:** Correlation between serum HE4 levels and clinicopathologic parameters in patients with ductal carcinoma in situ.

Characteristics (Number)	Serum HE4 Levels Mean ± S.D.	*p* Value
Menopause		< 0.05
Pre (27)	35.3 ± 6.4	
Post (32)	42.9 ± 14.4	
Size (cm)		0.881
<2.8 (36)	39.6 ± 12.8	
≥2.8 (23)	39.1 ± 10.8	
Nuclear grade		0.516
1 (1)	31.3	
2 (27)	37.9 ± 9.1	
3 (31)	40.9 ± 14.1	
Comedo-type necrosis		0.594
No (10)	37.5 ± 10.3	
Yes (49)	39.8 ± 12.3	
Stromal TILs density score		0.716
1 (39)	40.3 ± 11.6	
2 (19)	37.6 ± 13.0	
3 (1)	41.7	
Estrogen receptor-α		0.868
Negative (25)	39.1 ± 14.5	
Positive (34)	39.7 ± 9.9	
HER-2		0.692
Negative (31)	38.8 ± 11.5	
Positive (28)	40.1 ± 12.6	
Molecular subtypes		0.917
HR+/HER2− (27)	38.5 ± 10.3	
HR+/HER2+ (11)	41.2 ± 7.3	
HR−/HER2+ (17)	39.4 ±15.3	
Triple-negative (4)	41.4 ± 20.2	
Recurrence		0.222
No (53)	40.1 ± 12.3	
Yes (6)	33.8 ± 6.9	

S.D., standard deviation; ns, not significant; TILs, tumor-infiltrating lymphocytes.

**Table 2:** Crosstabulation of HE4 status by mRNA RNAscope in situ hybridization and protein immunohistochemistry in DCIS tissues.

RNAscope In Situ Hybridization	Immunohistochemistry		Total	Concordance	*κ* Value	*p* Value
	Low	High				
Low	64	10	77	88.9	0.658	<0.001
High	1	24	25			
Total	65	34	99			

**Table 3:** Relationship between HE4 mRNA and protein expression and clinicopathologic parameters in ductal carcinoma in situ.

Characteristics	High mRNA HE4 Expression *N*/Total *N* (%)	*p* Value	High Protein HE4 Expression *N*/Total *N* (%)	*p* Value
Menopause		0.525		0.942
Pre	14/53 (26.4)		18/53 (34.0)	
Post	11/46 (23.9)		16/46 (34.8)	
Size (cm)		0.689		0.108
<2.8	15/56 (26.8)		23/56 (41.1)	
≥2.8	10/43 (23.3)		11/43 (25.6)	
Nuclear grade		0.129		0.068
1	1/3 (33.3)		1/3 (33.3)	
2	17/55 (30.9)		25/55 (45.5)	
3	7/41 (17.1)		8/41 (19.5)	
Comedo-type necrosis		0.080		<0.05
No	9/23 (39.1)		13/23 (56.5)	
Yes	16/76 (21.1)		21/76 (27.6)	
Stromal TILs density score		<0.05		<0.05
1	22/67 (32.8)		28/67 (41.8)	
2	3/30 (10.0)		6/30 (20.0)	
3	0/2 (0)		0/2 (0)	
Estrogen receptor-α		<0.01		<0.001
Negative	4/40 (10.0)		5/40 (12.5)	
Positive	21/59 (35.6)		29/59 (49.2)	
HER-2		<0.05		<0.001
Negative	18/54 (33.3)		27/54 (50.0)	
Positive	7/45 (15.6)		7/45 (15.6)	
Molecular subtypes		0.099		<0.01
HR+/HER2−	17/48 (35.4)		25/48 (52.1)	
HR+/HER2+	4/16 (25.0)		4/16(25.0)	
HR−/HER2+	3/29 (10.3)		3/29 (10.3)	
Triple-negative	1/6 (16.7)		2/6 (33.3)	
Recurrence		0.688		0.489
No	22/90 (24.4)		30/90 (33.3)	
Yes	3/9 (33.3)		4/9 (44.4)	

*N*, number; TILs, tumor-infiltrating lymphocytes.

## Data Availability

The data presented in this study are available on request from the corresponding author.

## Abbreviations

The following abbreviations are used in this manuscript:

HE4	Human epididymis protein 4
DCIS	Ductal carcinoma in situ
ER	Estrogen receptor
PR	Progesterone receptor
HER2	Human epidermal growth factor receptor 2
HR	Hormone receptor
WAP	Whey acidic protein
ISH	In situ hybridization
FFPE	Formalin-fixed-paraffin-embedded
CNUHH	Chonnam National University Hwasun Hospital
TILs	Tumor-infiltrating lymphocytes
